# Nucleosome DNA sequence structure of isochores

**DOI:** 10.1186/1471-2164-12-203

**Published:** 2011-04-21

**Authors:** Zakharia M Frenkel, Thomas Bettecken, Edward N Trifonov

**Affiliations:** 1 Genome Diversity Center, Institute of Evolution, University of Haifa, Mount Carmel, Haifa 31905, Israel; 2 CAGT-Center for Applied Genotyping, Max Planck Institute of Psychiatry, Kraepelinstr. 2-10, D-80804 Muenchen, Germany; 3 Department of Functional Genomics and Proteomics, Faculty of Science, Masaryk University, Kotlarska 2, CZ-61137 Brno, Czech Republic

## Abstract

**Background:**

Significant differences in G+C content between different isochore types suggest that the nucleosome positioning patterns in DNA of the isochores should be different as well.

**Results:**

Extraction of the patterns from the isochore DNA sequences by Shannon N-gram extension reveals that while the general motif YRRRRRYYYYYR is characteristic for all isochore types, the dominant positioning patterns of the isochores vary between TAAAAATTTTTA and CGGGGGCCCCCG due to the large differences in G+C composition. This is observed in human, mouse and chicken isochores, demonstrating that the variations of the positioning patterns are largely G+C dependent rather than species-specific. The species-specificity of nucleosome positioning patterns is revealed by dinucleotide periodicity analyses in isochore sequences. While human sequences are showing CG periodicity, chicken isochores display AG (CT) periodicity. Mouse isochores show very weak CG periodicity only.

**Conclusions:**

Nucleosome positioning pattern as revealed by Shannon N-gram extension is strongly dependent on G+C content and different in different isochores. Species-specificity of the pattern is subtle. It is reflected in the choice of preferentially periodical dinucleotides.

## Background

The nucleosome positioning signal in human genome sequences is rather weak. It lacks the periodical AA and TT dinucleotides, the main component of the nucleosome positioning pattern in most of other genomes [1,2]. Similarly, the mouse genome is featureless in terms of dinucleotide periodicities [2]. This lack of periodicities, diagnostic of the presence of a nucleosome positioning signal, makes the extraction of a nucleosome signal from such "silent" genomes problematic. One possible way to tackle this problem is to analyze the oligonucleotide composition of DNA sequences, which may reflect to some degree the hidden positioning patterns. The pattern-specific short oligonucleotides would be expected to appear more often in the overall vocabularies of the oligonucleotides, which then may be used for detection of the pattern. Indeed, recent Shannon N-gram extension analysis [3] of eukaryotic genomes [4] revealed that the majority of the genomes are characterized by the same hidden sequence motif GRAAATTTYC which, according to latest studies, represents the nucleosome positioning DNA bendability pattern [5-7].

It is known for many years that the genomes of warm blooded vertebrates are organized into regions of rather uniform G+C content, termed isochores [8]. The regional base composition of the isochores exerts pressure on all kinds of sequences within the isochores, and on all three positions of the codons in the protein coding sequences [8]. Many genomic features and functions are influenced by the G+C content, such as gene density, activity of the genes, timing of replication, recombination events and others [8-10]. It seems therefore natural, to calculate di- and oligonucleotide periodicities in the isochore subfractions of different genomes and compare the results. There are five major isochore types, L1, L2, H1, H2 and H3, with G+C content varying between about <37% (L1) and >52% (H3). The standard nucleosome pattern, GRAAATTTYC, is an average motif to characterize a whole genome. One would expect that higher isochores, with reduced content of AA and TT dinucleotides, would have rather different, more G+C-rich nucleosome positioning pattern. The other extreme, isochores L1 and L2, would likely be characterized by an A+T-rich positioning pattern. It has been reported that the nucleosome formation potential is higher in A+T-rich isochores [11]. That suggests that the AA and TT elements of the pattern, perhaps, are the strongest contributors for nucleosome formation. This is also consistent with positional autocorrelation data [2]. In this study, a large scale analysis of di- and oligonucleotide periodicities in five types of isochore sequences, both in humans and in mice [9,10] and in six types of isochore sequences in chicken [12] is performed. Apart from differences in G+C composition [9], and di- and trinucleotide composition [13], the isochores appear to be different in terms of the dominant N-gram extension motifs, suggesting significant differences in their nucleosome positioning patterns. The analysis of the isochore sequences suggests that the calculated positioning patterns have both strong isochore-specific components (G+C rich and A+T rich motifs) and species-specific components, reflecting different usage of periodically positioned dinucleotides.

## Results and Discussion

### Sequence periodicities in isochores

**Figure 1:**
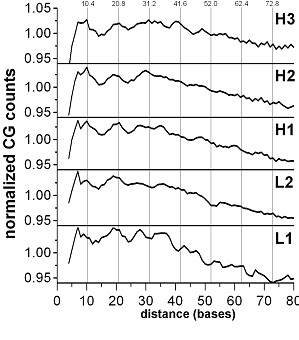
**Positional autocorrelation of CG dinucleotides in different isochores of the human genome**. The normalized histograms of occurrences of the dinucleotide pairs at distances 2-80 bases from one another are shown. The histograms are smoothed by running average of 3 positions. The level 1.00 corresponds to the average counts within the interval 0-80 (53.5 × 10^3 ^for H3, 92.0 × 10^3 ^for H2, 74.8 × 10^3 ^for H1, 45.5 × 10^3 ^for L2 and 9.5 × 10^3 ^for L1). The 10.4 base periodical distances are shown by vertical bars.

In the human genome, the only dinucleotide that shows a clear 10.4 base periodicity is CG [2]. The periodicity plots calculated separately for all five types of human isochores are shown in Figure 1. The sequences with repeats masked are used in all cases. The occurrence of CG dinucleotides is higher in G+C rich isochores, which is not surprising. The ~10.4 base periodicity of CG dinucleotides also shows an increase in visibility when moving from L1 to H3. In the periodicity plot for H3, four maxima are seen, at positions ~10, 21, 31 and 41 - the nearest integers to multiples of 10.4 bases (10.4, 20.8, 31.2, 41.6 bases). The number of visible peaks decreases towards the lightest isochores L2 (peaks 10, 20, 30 for H2; 10, 20 - for H1; and only a peak at ~20 is visible in plots for L1 and L2). Periodicities of other dinucleotides are not detectable in human isochores this way, confirming earlier results [2].

**Figure 2:**
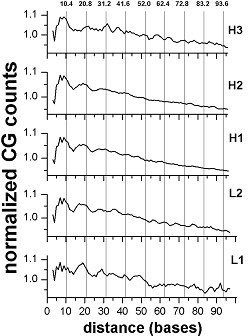
**Positional autocorrelation of CG dinucleotides in different isochores of the mouse genome**. The level 1.0 corresponds to 3.6 × 10^3 ^for H3, 90.1 × 10^3 ^for H2, 101.7 × 10^3 ^for H1, 30.6 × 10^3 ^for L2, 2.4 × 10^3 ^for L1 (for further details see legend to Figure 1)

Similar distance analyses applied to the isochores of mouse did not reveal any strong 10-11 base periodicities, as one would expect from the whole mouse genome data (ibid). However, CG does show a weak periodicity in some of the mouse isochores (Figure 2). From one to three peaks, at positions close to multiples of 10.4 bases, are seen, with increasing amplitude towards H3.

**Figure 3:**
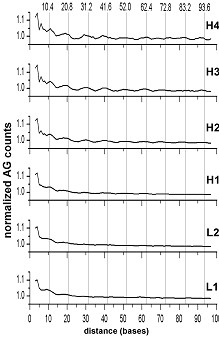
**Positional autocorrelation of AG dinucleotides in different isochores of the chicken genome**. The level 1.0 corresponds to 19.8 × 10^3 ^for H4, 100.6 × 10^3 ^for H3, 640.1 × 10^3 ^for H2, 1471.9 × 10^3 ^for H1, 1768.4 × 10^3 ^for L2, 572.1 × 10^3 ^for L1 (for further details see legend to Figure 1)

The chicken isochores, in full accordance with earlier whole genome data (ibid), manifest periodicity for the AG dinucleotide, increasing as well when moving from L1 to H3 (Figure 3).

### Variations of nucleosome positioning pattern in isochores

Application of the N-gram (trinucleotide) extension procedure [3,4] to the isochore sequences has proven to yield very informative results. Obviously, various patterns carried by the sequences are reflected in oligonucleotide (N-gram) frequencies, especially those patterns that are dominant in the genomes, like the nucleosome positioning motif GRAAATTTYC [5]. The trinucleotides of which this pattern consists (GRA, RAA, AAA, AAT,...) do appear in the sequences more often, so that just inspection of the top scoring triplets already gives a fair idea about the hidden pattern. The motif in its entirety practically does not appear in the genomes, with the exception of C. elegans [7]. This, perhaps, can be explained by avoidance of very strong nucleosomes as they may be an obstacle for replication and transcription. Besides, too strong adherence of sequences to any particular pattern would prevent other messages to be coded in the same sequences. It is known that the genomic sequences carry multiple overlapping codes coexisting due to their degeneracy [14,15]. For example, exons and splice junction sequences often reside in nucleosomes [16,17], which means that at least three different codes can overlap on the same sequence.

Application of the Shannon N-gram extension to human, mouse and chicken genomes reveals that these and other genomes possess the same overall dominant pattern GRAAATTTYC [4]. It is expressed in the highest occurrences of its component trinucleotides in respective N-gram tables. The same analysis, applied separately to different types of isochores of the above three species, shows that the N-gram extensions for different isochores result in rather diverse patterns. The analysis described below is performed on the isochore sequences with masked repeats. Comparison of the N-gram tables for the masked isochore sequences (Additional file 1, Table S1) revealed that the trinucleotides in this table follow the same sorting order as for N-grams of the complete genome isochores, without discarding the repeats [13], at least within the top 20 ranks.

Starting with TTT, the most frequent triplet in human isochores L1 (Additional file 1, Table S1), one derives the pattern [(A)(T)](A)(T)[(A)(T)] with AT-central AAATTT (or AAAATTTT, or AAAAATTTTT) in the middle. Here, parentheses correspond to an uncertain number of repetitions of the bases (motifs) included in them. The most frequent CG containing triplet, ACG, extends to [(T)(A)](T)(A)CG(T)(A)[(T)(A)] that does not match to the AT-central motifs above. However, upstream and downstream from the rare triplets ACG and CGT in this expression, the motif (A)(T) takes over. The same is observed for the human isochores L2.

The topmost triplet TTT of the isochores H1 extends to complementarily symmetrical AT-central (CA)CAG(A)(T)CTG(TG), while the extension from CGG generates (CA)CAG(A)(T)CCGG(A)(T)CTG(TG), CG-central, with two almost exact copies of the above AT-central motif in the non-repetitive middle.

Similarly constructed patterns for H2 isochores with higher G+C content are T(C)A(G), with the seed triplet CAG, and T(C)(G)A, with the seed triplet CCG. For H3 isochores, the reconstructed extension motifs are: A(G)(C)T (the topmost seed GGG) and A(G)(C)(G)(C)T (seed CGG).

**Figure 4:**
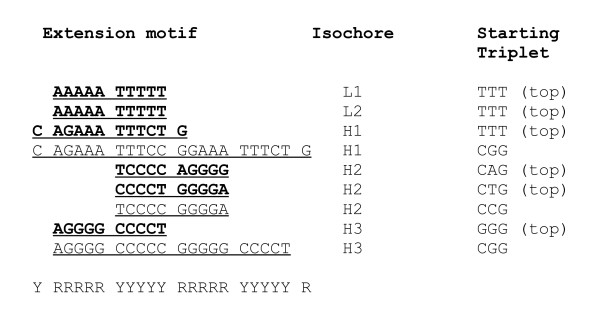
**Alignment of triplet extension patterns derived for the various types of human isochores**. The patterns constructed from the most frequent triplets are shown in bold.

The motifs described above may correspond to nucleosome positioning pattern only if the number of consecutive purines (A and G) does not exceed five residues. The same holds for pyrimidines (C and T). That feature of the nucleosome positioning motifs has been established in previous studies [18-20,6]. This removes the uncertainties in the repeat lengths of (A), (C), (G) and (T) in the sequence expressions above. The extension motifs of the isochores adjusted to the positioning pattern RRRRRYYYYY are shown in Figure 4.

Thus, the extension motifs are consistent with their possible nucleosome positioning function. Only the patterns derived for isochores H1 (with a G+C composition close to the average for the human genome), with the consensus RGAAATTTCY, resemble the nucleosome positioning standard GRAAATTTYC [5,6]. Others diverge from it in two opposite directions towards higher A+T or G+C content, all conforming, however, to the RR/YY pattern. In G+C rich isochores, the AT element of the standard may thus be replaced by GC, while the CG dinucleotide may be replaced by CA, TG and TA, respectively, in A+T rich isochores.

The results described above suggest that anomalously G+C rich or A+T rich sequences (parts of genomes or whole genomes) would have, respectively, deviant nucleosome positioning patterns, up to extremes (AAAAATTTTT)_n _and (GGGGGCCCCC)_n_, with the whole-genome averages typically approaching the standard (GRAAATTTYC)_n_.

**Figure 5:**
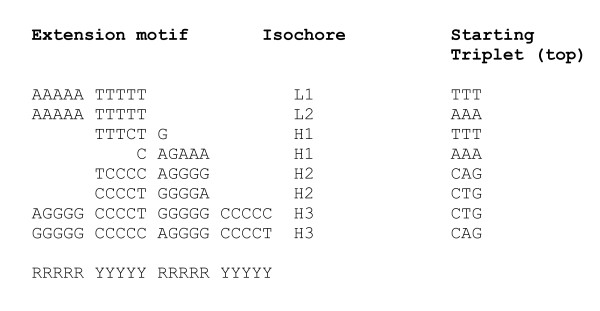
**Alignment of triplet extension patterns derived for the various types of mouse isochores**. The patterns are constructed from the most frequent triplets.

The same oligonucleotide extension analysis applied to the isochores of mouse is arriving at similar patterns, shown in Figure 5. Here as well, the common RRRRRYYYYY motif ranges between AAAAATTTTT and GGGGGCCCCC. Topmost triplets of mouse isochores H3 do not extend to a unique complementary symmetrical motif as in other isochore types. Instead, two motifs are generated starting from topmost TTT and AAA triplets. Both are parts of the standard RRRRRYYYYY motif, complementarily symmetrical to one another (Figure 5). The dominant patterns derived for different isochores, thus, suggest that depending on the G+C content different sequences may have different dominant nucleosome positioning motifs, with different usage of dinucleotides, while maintaining a similar degree of positioning or packaging of DNA into chromatin.

**Figure 6:**
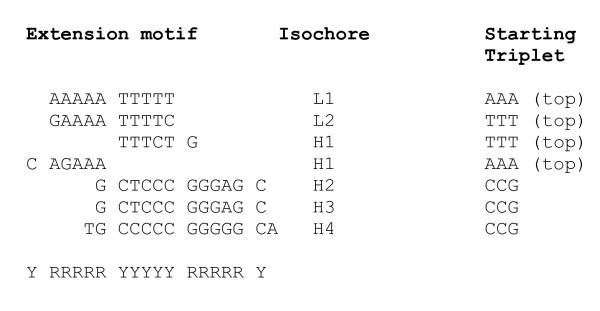
Alignment of triplet extension patterns derived for the various types of chicken isochores

The oligonucleotide extension analysis applied to the isochores of chicken result in patterns shown in Figure 6. Here as well, the extension motif for the isochores H1 is split in two, as in mouse.

**Figure 7:**
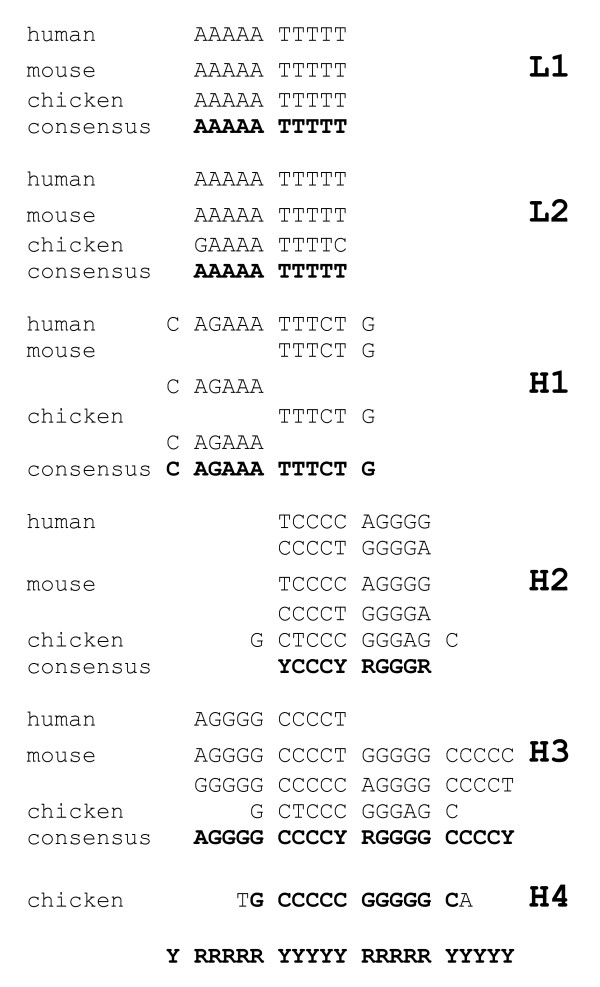
Comparison of the dominant extension patterns of isochores of three different species

The Shannon N-gram extension of isochores of three different species results in essentially identical patterns for isochores of the same type (Figure 7). The patterns vary between AAAAA TTTTT for isochores L1 and L2, and GGGGG CCCCC for isochores H3 and H4. Patterns for isochores H1 and H2, intermediate in terms of G+C composition, are intermediate as well.

## Conclusions

There are several different ways to derive the nucleosome positioning pattern from a given genome (chromosome, isochore) sequence - positional auto- and cross-correlation [21,20], signal regeneration [5], and N-gram extension [4]. Since the signal in most cases is very weak, some of the approaches may not be successful. The pattern extension approach suggests the most likely pattern for a given sequence, while ignoring less probable extensions. It may well be that the standard GRAAATTTYC is, actually, present in the extreme cases of isochores L1 and H3 as well, though at lower proportions. The final patterns which are representing an average rather than the most typical motifs for sequences of interest, would be obtained by derivation of complete matrices of bendability. The fact that even "canonical" AA and TT dinucleotides of the standard pattern do not manifest detectable periodicity neither in human nor in mouse genomes, means that these dinucleotides are not a frequent choice in the respective nucleosomes [2]. More often other well deformable elements (GG, CC, and, especially, CG) of the standard pattern are used. Similarly, the AG (CT) dinucleotide, at odds with the standard pattern, is more often used in chicken nucleosomes ([2], see also Figure 6).

The extension patterns obtained with our calculations indicate what would be the predominant dinucleotide elements in the respective matrices. In any case, the patterns above suggest significant differences of the bendability matrices depending on the isochore type. In particular, the additional dinucleotides which do not appear in the standard pattern GRAAATTTYC, namely, CA, TA, TG, AG, CT and GC, may well, indeed, be part of the nucleosome positioning signal and appear in the final matrices of bendability.

The variation of the nucleosome positioning pattern in isochores from A5T5 to G5C5 while keeping conformity to the R5Y5 pattern attests to importance of the alternating binary pattern RR/YY [20] and, apparently, less crucial role of the binary pattern SS/WW [22]. This also suggests that the stacking interactions between purines in the RR•YY stacks [6], and preferential roll-wise deformation of the RY•RY and YR•YR stacks [23,19] are major contributors to deformational anisotropy of DNA [24]. The preference of [A,T] base pair stacks to the minor grooves of the nucleosome DNA oriented outwards, as compared to [G,C] stacks [6], becomes essential for DNA with non-extreme (A+T)/(G+C) ratios.

## Methods

Complete human and mouse genome sequences were taken from http://hgdownload.cse.ucsc.edu/goldenPath/hg18/ and http://hgdownload.cse.ucsc.edu/goldenPath/mm9/, correspondingly, after repeat masking with the software RepeatMasker and Tandem Repeats Finder (with periods of 12 bases or less). These sequences have been assembled by the International Human Genome Project sequencing centers (hg18 in March 2006, mm9 in July 2007).

All programs used for DNA sequence analysis are written in C++ and are original. To exclude the end effects of short range distances in positional correlation analyses, the last dinucleotides at the ends (within the window size region) were not considered.

The selection of isochores was carried out according to [9,10], by using a window size of 100,000 bases.

Derivation of the patterns by the N-gram extension method [3] was performed as follows. The most frequent triplets in eukaryotic genomes are, typically, AAA, TTT, ATT, AAT, GAA, TTC,... (Additional file 1, Tables S1, S2 and S3). The extension motifs can be assembled by fusing a triplet ABC with the most frequent triplet of xAB family (upstream extension) and the most frequent triplet of BCx family (downstream). Extending the TTT triplet, thus, would result in the sequence T_n_, or (T), in the notation used in the paper for repetitions with uncertain number of repeats. If all continuations of the (T) string are performed with respective probabilities of other xTT and TTx triplets, the repeating (T) will continue in A(T)C = ATT...TTC, as both ATT and TTC triplets are among the most frequent ones. Further continuation with the most likely extension results, for example, in case of human isochore H1 (Figure 4) in the expression (CA)CAG(A)(T)CTG(TG), where the underlined sequence corresponds to the unique non-repeating middle part of the extension.

## List of Abbreviations

A: Adenine; C: Cytosine; G: Guanine; T: Thymidine; R: Purine (A or G); Y: Pyrimidine (C or T).

## Authors' contributions

ZMF authored code, did part of the calculations and analyses, contributed to the interpretation of the data and helped in drafting the manuscript. TB initiated the work, authored code, did part of the calculations and analyses, contributed to the interpretation of the data and edited the manuscript. ENT conceived the study, did part of the analyses contributed to the interpretation of the data and drafted the manuscript. All authors read and approved the manuscript.

## Supplementary Material

Additional file 1 **Table S1 - Trinucleotide frequencies in the human genome**. The table contains the list of all 64 possible trinucleotides in the human genome, ordered by their frequency in the respective isochore L1, L2, H1, H2 and H3. **Table S2 - Trinucleotide frequencies in the mouse genome**. The table contains the list of all 64 possible trinucleotides in the mouse genome, ordered by their frequency in the respective isochore L1, L2, H1, H2 and H3. **Table S3 - Trinucleotide frequencies in the chicken genome**. The table contains the list of all 64 possible trinucleotides in the chicken genome, ordered by their frequency in the respective isochore L1, L2, H1, H2, H3 and H4. Click here for file
