# Supplementary material for: Nucleosome DNA sequence structure of isochores
Source: BMC Genomics. 2011 Apr 21;12:203. doi: 10.1186/1471-2164-12-203 (PMC3097165; doi:10.1186/1471-2164-12-203)
Supplement: Additional file 1 — Table S1 - Trinucleotide frequencies in the human genome. The table contains the list of all 64 possible trinucleotides in the human genome, ordered by their frequency in the respective isochore L1, L2, H1, H2 and H3. Table S2 - Trinucleotide frequencies in the mouse genome. The table contains the list of all 64 possible trinucleotides in the mouse genome, ordered by their frequency in the respective isochore L1, L2, H1, H2 and H3. Table S3 - Trinucleotide frequencies in the chicken genome. The table contains the list of all 64 possible trinucleotides in the chicken genome, ordered by their frequency in the respective isochore L1, L2, H1, H2, H3 and H4. [file 1471-2164-12-203-S1.DOC]

**Additional file 1**

**The additional file 1 contains the tables of all 64 possible dinucleotides in the human (Table S1), the mouse (Table S2) and the chicken (Table S3) genome, ordered by their frequency and in the respective isochore.**

**Table S1 – Trinucleotide frequencies in the isochores of the human genome**

The table contains the list of all 64 possible trinucleotides in the human genome, ordered by their frequency in the respective isochore L1, L2, H1, H2 and H3.

|  | **Total** | | | **H3** | | | **H2** | | | **H1** | | | **L2** | | | **L1** | | |
| --- | --- | --- | --- | --- | --- | --- | --- | --- | --- | --- | --- | --- | --- | --- | --- | --- | --- | --- |
|  | triplet | amount | % | triplet | amount | % | triplet | amount | % | triplet | amount | % | triplet | amount | % | triplet | amount | % |
| 1 | TTT | 55463500 | 3.81 | GGG | 1759990 | 3.7 | CAG | 5425770 | 2.8 | TTT | 12511300 | 3.41 | TTT | 21627100 | 4.22 | TTT | 16277900 | 4.85 |
| 2 | AAA | 55261100 | 3.79 | CCC | 1753380 | 3.68 | CTG | 5425370 | 2.8 | AAA | 12434800 | 3.39 | AAA | 21557400 | 4.2 | AAA | 16255800 | 4.84 |
| 3 | ATT | 37827800 | 2.6 | CTG | 1588130 | 3.33 | CCC | 4864090 | 2.51 | TCT | 8377010 | 2.28 | ATT | 14786500 | 2.88 | ATT | 11736300 | 3.49 |
| 4 | AAT | 37766100 | 2.59 | CAG | 1587490 | 3.33 | GGG | 4849500 | 2.5 | AGA | 8357910 | 2.28 | AAT | 14761600 | 2.88 | AAT | 11734400 | 3.49 |
| 5 | TCT | 32877900 | 2.26 | CCT | 1390660 | 2.92 | CCT | 4840110 | 2.49 | ATT | 8184280 | 2.23 | TTA | 12833500 | 2.5 | TTA | 10195700 | 3.03 |
| 6 | AGA | 32831700 | 2.25 | AGG | 1390300 | 2.92 | AGG | 4826550 | 2.49 | AAT | 8162030 | 2.23 | TAA | 12813700 | 2.5 | TAA | 10194600 | 3.03 |
| 7 | TTA | 32606600 | 2.24 | GGC | 1361510 | 2.86 | CCA | 4603680 | 2.37 | CTG | 8029160 | 2.19 | TCT | 11911900 | 2.32 | TAT | 10005400 | 2.98 |
| 8 | TAA | 32566000 | 2.23 | GCC | 1360390 | 2.86 | TGG | 4600530 | 2.37 | CAG | 7996280 | 2.18 | TAT | 11902800 | 2.32 | ATA | 10003600 | 2.98 |
| 9 | CTT | 31097900 | 2.13 | TGG | 1323650 | 2.78 | TTT | 4428130 | 2.28 | CTT | 7897300 | 2.15 | AGA | 11901700 | 2.32 | TCT | 7645930 | 2.27 |
| 10 | AAG | 31036200 | 2.13 | CCA | 1318600 | 2.77 | AAA | 4394700 | 2.26 | AAG | 7871010 | 2.15 | ATA | 11890600 | 2.32 | AGA | 7634310 | 2.27 |
| 11 | TTC | 30802000 | 2.11 | GAG | 1159590 | 2.43 | CTC | 4215080 | 2.17 | TTC | 7699830 | 2.1 | CTT | 11455800 | 2.23 | TTC | 7541870 | 2.24 |
| 12 | GAA | 30756700 | 2.11 | CTC | 1156100 | 2.43 | GAG | 4213880 | 2.17 | GAA | 7693270 | 2.1 | AAG | 11441300 | 2.23 | GAA | 7530020 | 2.24 |
| 13 | TAT | 30258000 | 2.07 | GGA | 1102630 | 2.31 | TCT | 4103910 | 2.11 | TGT | 7248020 | 1.98 | TTC | 11401400 | 2.22 | CTT | 7356160 | 2.19 |
| 14 | ATA | 30233500 | 2.07 | TCC | 1097210 | 2.3 | AGA | 4096900 | 2.11 | ACA | 7177600 | 1.96 | GAA | 11387500 | 2.22 | AAG | 7348370 | 2.19 |
| 15 | TGT | 29098800 | 2 | CAC | 1022770 | 2.15 | TCC | 4051500 | 2.09 | TGA | 7151430 | 1.95 | TGT | 10519400 | 2.05 | TGT | 7100660 | 2.11 |
| 16 | CTG | 28971900 | 1.99 | GTG | 1020290 | 2.14 | GGA | 4046670 | 2.08 | TCA | 7129510 | 1.94 | ACA | 10482700 | 2.04 | ACA | 7090420 | 2.11 |
| 17 | ACA | 28966600 | 1.99 | AGC | 1008540 | 2.12 | GCC | 3769400 | 1.94 | CCT | 7081350 | 1.93 | TGA | 10398500 | 2.03 | TCA | 6926960 | 2.06 |
| 18 | CAG | 28931100 | 1.98 | GCT | 1002790 | 2.1 | GGC | 3758640 | 1.93 | AGG | 7072460 | 1.93 | TCA | 10394300 | 2.02 | TGA | 6914910 | 2.06 |
| 19 | TGA | 28583600 | 1.96 | GCA | 964467 | 2.02 | CTT | 3682830 | 1.9 | TTA | 7002520 | 1.91 | TTG | 10064100 | 1.96 | CAT | 6880420 | 2.05 |
| 20 | TCA | 28569600 | 1.96 | TGC | 959665 | 2.01 | AAG | 3668890 | 1.89 | TAA | 6986070 | 1.9 | CAA | 10028500 | 1.95 | ATG | 6875170 | 2.04 |
| 21 | CAT | 27168400 | 1.86 | AGA | 840839 | 1.76 | AGC | 3566470 | 1.84 | TGG | 6945600 | 1.89 | CAT | 10016800 | 1.95 | TTG | 6782500 | 2.02 |
| 22 | ATG | 27159800 | 1.86 | TCT | 839120 | 1.76 | GCT | 3562190 | 1.83 | CCA | 6894270 | 1.88 | ATG | 10014200 | 1.95 | CAA | 6777070 | 2.02 |
| 23 | TTG | 27092800 | 1.86 | ACC | 780718 | 1.64 | GTG | 3535250 | 1.82 | TTG | 6718590 | 1.83 | CTG | 9065960 | 1.77 | AGT | 5836070 | 1.73 |
| 24 | CAA | 26959400 | 1.85 | GGT | 780653 | 1.64 | CAC | 3530050 | 1.82 | ATG | 6666420 | 1.82 | CAG | 9054310 | 1.76 | ACT | 5834340 | 1.73 |
| 25 | CCT | 25469900 | 1.75 | ACA | 756616 | 1.59 | TTC | 3504910 | 1.8 | CAT | 6659630 | 1.81 | AGT | 8755600 | 1.7 | GTT | 5428770 | 1.61 |
| 26 | AGG | 25439200 | 1.74 | TGT | 754045 | 1.58 | GAA | 3488100 | 1.8 | CAA | 6647900 | 1.81 | ACT | 8746050 | 1.7 | AAC | 5419940 | 1.61 |
| 27 | TGG | 25064400 | 1.72 | TGA | 708625 | 1.49 | TGT | 3476670 | 1.79 | GAG | 6415770 | 1.75 | GTT | 8056500 | 1.57 | CTA | 5059520 | 1.5 |
| 28 | CCA | 24998800 | 1.71 | TCA | 707127 | 1.48 | TGC | 3465980 | 1.78 | CTC | 6398800 | 1.74 | AAC | 8027160 | 1.56 | TAG | 5054840 | 1.5 |
| 29 | AGT | 23659100 | 1.62 | AAG | 706595 | 1.48 | GCA | 3461110 | 1.78 | GGA | 6252310 | 1.7 | CCT | 7965450 | 1.55 | CAG | 4867250 | 1.45 |
| 30 | ACT | 23620200 | 1.62 | CTT | 705852 | 1.48 | ACA | 3459290 | 1.78 | TCC | 6237170 | 1.7 | AGG | 7963770 | 1.55 | CTG | 4863240 | 1.44 |
| 31 | GAG | 23149800 | 1.59 | GAA | 657824 | 1.38 | TCA | 3411690 | 1.76 | TAT | 6189750 | 1.69 | TGG | 7944670 | 1.55 | ATC | 4734340 | 1.41 |
| 32 | CTC | 23127100 | 1.58 | TTC | 653955 | 1.37 | TGA | 3410210 | 1.76 | ATA | 6180230 | 1.68 | CCA | 7925590 | 1.54 | GAT | 4729240 | 1.4 |
| 33 | GGA | 22516600 | 1.54 | GAC | 642919 | 1.35 | CAT | 3025690 | 1.56 | AGT | 5861070 | 1.6 | GAG | 7371670 | 1.43 | TAC | 4607230 | 1.37 |
| 34 | TCC | 22499300 | 1.54 | GTC | 642146 | 1.35 | ATG | 3020490 | 1.55 | ACT | 5837210 | 1.59 | CTC | 7362910 | 1.43 | GTA | 4602820 | 1.37 |
| 35 | GTT | 21572900 | 1.48 | TTT | 619156 | 1.3 | TTG | 2973740 | 1.53 | TGC | 5600170 | 1.53 | TAG | 7245100 | 1.41 | CCA | 4256620 | 1.26 |
| 36 | AAC | 21477500 | 1.47 | AAA | 618439 | 1.3 | CAA | 2955740 | 1.52 | GCA | 5582470 | 1.52 | CTA | 7236640 | 1.41 | TGG | 4249990 | 1.26 |
| 37 | TGC | 20742000 | 1.42 | CAT | 585924 | 1.23 | ATT | 2759040 | 1.42 | GGG | 5558160 | 1.51 | GGA | 7230180 | 1.41 | CCT | 4192380 | 1.24 |
| 38 | GCA | 20716700 | 1.42 | ATG | 583462 | 1.22 | ACC | 2758100 | 1.42 | GTG | 5541430 | 1.51 | TCC | 7225340 | 1.41 | AGG | 4186130 | 1.24 |
| 39 | GTG | 20327400 | 1.39 | TTG | 553819 | 1.16 | GGT | 2754550 | 1.42 | CCC | 5531780 | 1.51 | GAT | 7044060 | 1.37 | CTC | 3994190 | 1.19 |
| 40 | CAC | 20255200 | 1.39 | CAA | 550193 | 1.15 | AAT | 2746850 | 1.41 | GCT | 5525610 | 1.51 | ATC | 7034510 | 1.37 | GAG | 3988860 | 1.18 |
| 41 | GCT | 20135200 | 1.38 | CGG | 531161 | 1.11 | AGT | 2677750 | 1.38 | AGC | 5508620 | 1.5 | TGC | 6783230 | 1.32 | TGC | 3932910 | 1.17 |
| 42 | AGC | 20124000 | 1.38 | CCG | 529285 | 1.11 | ACT | 2673970 | 1.38 | CAC | 5490590 | 1.5 | GCA | 6778010 | 1.32 | GCA | 3930620 | 1.17 |
| 43 | GGG | 19509100 | 1.34 | ACT | 528636 | 1.11 | GTC | 2327810 | 1.2 | GTT | 5337310 | 1.45 | GTG | 6526850 | 1.27 | TCC | 3888100 | 1.15 |
| 44 | CCC | 19483700 | 1.33 | AGT | 528629 | 1.11 | GAC | 2327260 | 1.2 | AAC | 5292110 | 1.44 | CAC | 6505810 | 1.27 | GGA | 3884750 | 1.15 |
| 45 | GAT | 19079100 | 1.31 | GTT | 430532 | 0.9 | GTT | 2319820 | 1.19 | GAT | 4734230 | 1.29 | GCT | 6471140 | 1.26 | CAC | 3706030 | 1.1 |
| 46 | ATC | 19051000 | 1.3 | AAC | 430062 | 0.9 | AAC | 2308240 | 1.19 | ATC | 4714620 | 1.28 | AGC | 6464270 | 1.26 | GTG | 3703630 | 1.1 |
| 47 | TAG | 19006500 | 1.3 | GCG | 413450 | 0.86 | TTA | 2284450 | 1.17 | TAG | 4529460 | 1.23 | GTA | 6348370 | 1.23 | AGC | 3576080 | 1.06 |
| 48 | CTA | 18976000 | 1.3 | ATC | 411936 | 0.86 | TAA | 2281480 | 1.17 | CTA | 4508780 | 1.23 | TAC | 6347720 | 1.23 | GCT | 3573450 | 1.06 |
| 49 | GTA | 16565200 | 1.13 | GAT | 411920 | 0.86 | GAT | 2159690 | 1.11 | GGC | 4494050 | 1.22 | GGT | 5161920 | 1 | ACC | 2852610 | 0.85 |
| 50 | TAC | 16551400 | 1.13 | CGC | 411347 | 0.86 | ATC | 2155650 | 1.11 | GCC | 4490030 | 1.22 | ACC | 5149850 | 1 | GGT | 2847710 | 0.84 |
| 51 | GCC | 15935800 | 1.09 | ATT | 361666 | 0.76 | TAT | 1928580 | 0.99 | GGT | 4341640 | 1.18 | GGG | 5109310 | 0.99 | GTC | 2637410 | 0.78 |
| 52 | GGC | 15927100 | 1.09 | AAT | 361215 | 0.75 | ATA | 1925600 | 0.99 | ACC | 4317110 | 1.17 | CCC | 5097900 | 0.99 | GAC | 2633830 | 0.78 |
| 53 | GGT | 15886500 | 1.09 | TAG | 314414 | 0.66 | TAG | 1862720 | 0.96 | GTA | 3850360 | 1.05 | GTC | 4587360 | 0.89 | CCC | 2236550 | 0.66 |
| 54 | ACC | 15858400 | 1.09 | CTA | 310975 | 0.65 | CTA | 1860100 | 0.96 | TAC | 3836310 | 1.04 | GAC | 4579180 | 0.89 | GGG | 2232170 | 0.66 |
| 55 | GTC | 13967300 | 0.96 | TTA | 290498 | 0.61 | GTA | 1509890 | 0.77 | GTC | 3772560 | 1.03 | GGC | 4311970 | 0.84 | GCC | 2006150 | 0.59 |
| 56 | GAC | 13941500 | 0.95 | TAA | 290181 | 0.61 | TAC | 1507410 | 0.77 | GAC | 3758300 | 1.02 | GCC | 4309850 | 0.84 | GGC | 2000960 | 0.59 |
| 57 | CGG | 3840190 | 0.26 | CGT | 282548 | 0.59 | CCG | 1137720 | 0.58 | CGG | 1074060 | 0.29 | CGT | 1053330 | 0.2 | ACG | 562001 | 0.16 |
| 58 | CCG | 3837810 | 0.26 | ACG | 281723 | 0.59 | CGG | 1135600 | 0.58 | CCG | 1072810 | 0.29 | ACG | 1051680 | 0.2 | CGT | 561568 | 0.16 |
| 59 | CGT | 3705830 | 0.25 | GTA | 253805 | 0.53 | CGC | 914143 | 0.47 | CGT | 1032260 | 0.28 | TCG | 824897 | 0.16 | CGA | 421109 | 0.12 |
| 60 | ACG | 3697260 | 0.25 | TAC | 252779 | 0.53 | GCG | 912528 | 0.47 | ACG | 1027270 | 0.28 | CGA | 822205 | 0.16 | TCG | 420223 | 0.12 |
| 61 | GCG | 3149860 | 0.21 | ATA | 233455 | 0.49 | CGT | 776130 | 0.4 | GCG | 897275 | 0.24 | CGG | 800251 | 0.15 | CCG | 299683 | 0.08 |
| 62 | CGC | 3144180 | 0.21 | TAT | 231520 | 0.48 | ACG | 774587 | 0.39 | CGC | 892614 | 0.24 | CCG | 798308 | 0.15 | CGG | 299121 | 0.08 |
| 63 | TCG | 2924610 | 0.2 | TCG | 221542 | 0.46 | TCG | 632794 | 0.32 | TCG | 825155 | 0.22 | GCG | 675601 | 0.13 | CGC | 251218 | 0.07 |
| 64 | CGA | 2919470 | 0.2 | CGA | 221063 | 0.46 | CGA | 631574 | 0.32 | CGA | 823523 | 0.22 | CGC | 674853 | 0.13 | GCG | 251003 | 0.07 |

**Table S2 – Trinucleotide frequencies in the isochores of the mouse genome**

Trinucleotide frequencies in the mouse genome (without repeating sequences) and in the isochores L1, L2, H1, H2 and H3.

|  | **Total** | | | **H3** | | | **H2** | | | **H1** | | | **L2** | | | **L1** | | |
| --- | --- | --- | --- | --- | --- | --- | --- | --- | --- | --- | --- | --- | --- | --- | --- | --- | --- | --- |
|  | triplet | amount | % | triplet | amount | % | triplet | amount | % | triplet | amount | % | triplet | amount | % | triplet | amount | % |
| 1 | TTT | 48374200 | 3.38 | CTG | 183281 | 3.21 | CAG | 7092110 | 2.76 | TTT | 17408400 | 3.13 | TTT | 20041800 | 4 | TTT | 5265190 | 4.66 |
| 2 | AAA | 48355300 | 3.38 | CAG | 181905 | 3.19 | CTG | 7087920 | 2.76 | AAA | 17380200 | 3.13 | AAA | 20031800 | 4 | AAA | 5265190 | 4.66 |
| 3 | AGA | 33620800 | 2.35 | CCC | 167544 | 2.93 | CCT | 6082460 | 2.37 | TCT | 13201700 | 2.37 | ATT | 13990700 | 2.79 | AAT | 3932290 | 3.48 |
| 4 | TCT | 33617300 | 2.35 | GGG | 164035 | 2.87 | AGG | 6072200 | 2.36 | AGA | 13196200 | 2.37 | AAT | 13985300 | 2.79 | ATT | 3929180 | 3.48 |
| 5 | ATT | 32301100 | 2.25 | CCT | 160849 | 2.82 | AGA | 5873500 | 2.28 | CTG | 12384000 | 2.23 | TTA | 12354900 | 2.47 | ATA | 3546410 | 3.14 |
| 6 | AAT | 32296000 | 2.25 | AGG | 157937 | 2.77 | TCT | 5870390 | 2.28 | CAG | 12371600 | 2.22 | TAA | 12348800 | 2.46 | TAT | 3545780 | 3.14 |
| 7 | TGT | 31495500 | 2.2 | CCA | 150766 | 2.64 | CCA | 5812880 | 2.26 | TGT | 12235200 | 2.2 | TAT | 12174300 | 2.43 | TTA | 3399510 | 3.01 |
| 8 | ACA | 31452400 | 2.19 | TGG | 150191 | 2.63 | TGG | 5794680 | 2.25 | CTT | 12197500 | 2.19 | ATA | 12172800 | 2.43 | TAA | 3399450 | 3.01 |
| 9 | CTT | 31067600 | 2.17 | CTC | 134553 | 2.36 | AAA | 5600980 | 2.18 | ACA | 12188900 | 2.19 | AGA | 11872500 | 2.37 | TGT | 2585980 | 2.29 |
| 10 | AAG | 31062600 | 2.17 | GCC | 132634 | 2.32 | TTT | 5579870 | 2.17 | AAG | 12182900 | 2.19 | TCT | 11866300 | 2.37 | ACA | 2585280 | 2.29 |
| 11 | CTG | 30043200 | 2.1 | GAG | 132489 | 2.32 | CTC | 5482690 | 2.13 | TTC | 11310000 | 2.03 | TGT | 11294100 | 2.25 | AGA | 2564080 | 2.27 |
| 12 | CAG | 30035800 | 2.09 | GGC | 130555 | 2.29 | GAG | 5465750 | 2.13 | GAA | 11301200 | 2.03 | ACA | 11292400 | 2.25 | TCT | 2561550 | 2.27 |
| 13 | GAA | 29488900 | 2.06 | TCC | 124527 | 2.18 | CCC | 5462370 | 2.12 | ATT | 11035000 | 1.98 | AAG | 11168100 | 2.23 | GAA | 2531140 | 2.24 |
| 14 | TTC | 29485300 | 2.06 | GGA | 123182 | 2.16 | GGG | 5443940 | 2.12 | AAT | 11025500 | 1.98 | CTT | 11166000 | 2.23 | TTC | 2526640 | 2.23 |
| 15 | TTA | 29108600 | 2.03 | CAC | 120890 | 2.12 | ACA | 5277850 | 2.05 | TCA | 10906600 | 1.96 | GAA | 11001700 | 2.19 | ATG | 2479840 | 2.19 |
| 16 | TAA | 29096600 | 2.03 | AGC | 120448 | 2.11 | TGT | 5271000 | 2.05 | TGA | 10901400 | 1.96 | TTC | 10994400 | 2.19 | CAT | 2477250 | 2.19 |
| 17 | TCA | 28520500 | 1.99 | GCT | 120427 | 2.11 | AAG | 5180690 | 2.01 | AGG | 10369100 | 1.86 | TGA | 10451900 | 2.08 | AAG | 2433210 | 2.15 |
| 18 | TGA | 28512100 | 1.99 | GTG | 119729 | 2.1 | CTT | 5172430 | 2.01 | CCT | 10366800 | 1.86 | TCA | 10449500 | 2.08 | CTT | 2432000 | 2.15 |
| 19 | TAT | 27610400 | 1.93 | TCT | 117402 | 2.06 | TCC | 5035460 | 1.96 | CAT | 10350000 | 1.86 | ATG | 10418400 | 2.08 | TGA | 2423370 | 2.14 |
| 20 | ATA | 27606000 | 1.92 | AGA | 114569 | 2.01 | GGA | 5023600 | 1.95 | ATG | 10347200 | 1.86 | CAT | 10416300 | 2.08 | TCA | 2422200 | 2.14 |
| 21 | CAT | 27488100 | 1.92 | TGT | 109173 | 1.91 | AGC | 4870490 | 1.89 | TGG | 10223700 | 1.84 | TTG | 9865210 | 1.97 | TTG | 2295620 | 2.03 |
| 22 | ATG | 27487400 | 1.92 | TGC | 108798 | 1.9 | GCT | 4861270 | 1.89 | TTA | 10196300 | 1.83 | CAA | 9860660 | 1.97 | CAA | 2293560 | 2.03 |
| 23 | TTG | 26474700 | 1.85 | ACA | 107942 | 1.89 | CAC | 4799490 | 1.87 | CCA | 10195800 | 1.83 | AGT | 8848890 | 1.76 | ACT | 1998690 | 1.77 |
| 24 | CAA | 26439200 | 1.84 | GCA | 107057 | 1.87 | GTG | 4786910 | 1.86 | TAA | 10191100 | 1.83 | ACT | 8846890 | 1.76 | AGT | 1998600 | 1.77 |
| 25 | CCT | 25272400 | 1.76 | CTT | 99682 | 1.74 | TCA | 4648440 | 1.81 | TTG | 10148500 | 1.82 | CAG | 8782090 | 1.75 | GTT | 1861980 | 1.65 |
| 26 | AGG | 25267100 | 1.76 | AAG | 97706 | 1.71 | TGA | 4642350 | 1.8 | CAA | 10105000 | 1.81 | CTG | 8781120 | 1.75 | AAC | 1860110 | 1.64 |
| 27 | TGG | 24936600 | 1.74 | GGT | 95263 | 1.67 | GAA | 4572920 | 1.78 | GAG | 9827140 | 1.76 | GTT | 8063570 | 1.61 | TAG | 1710520 | 1.51 |
| 28 | CCA | 24922600 | 1.74 | ACC | 95201 | 1.67 | TTC | 4570230 | 1.78 | CTC | 9814160 | 1.76 | AAC | 8063250 | 1.61 | CTA | 1708420 | 1.51 |
| 29 | AGT | 24253200 | 1.69 | TCA | 93798 | 1.64 | GCA | 4414030 | 1.72 | AGT | 9402750 | 1.69 | TGG | 7407760 | 1.48 | ATC | 1627060 | 1.44 |
| 30 | ACT | 24240500 | 1.69 | TGA | 93053 | 1.63 | TGC | 4409340 | 1.71 | ACT | 9386550 | 1.69 | CCA | 7404600 | 1.48 | GAT | 1627040 | 1.44 |
| 31 | CTC | 23813700 | 1.66 | TTC | 83922 | 1.47 | GCC | 4324940 | 1.68 | TAT | 9146770 | 1.64 | TAG | 7361840 | 1.47 | GTA | 1608310 | 1.42 |
| 32 | GAG | 23806800 | 1.66 | GTC | 82785 | 1.45 | GGC | 4318450 | 1.68 | ATA | 9140010 | 1.64 | CTA | 7359130 | 1.47 | CAG | 1608100 | 1.42 |
| 33 | GGA | 22236500 | 1.55 | GAC | 82293 | 1.44 | CAT | 4164690 | 1.62 | GGA | 9117910 | 1.64 | AGG | 7340930 | 1.46 | TAC | 1606990 | 1.42 |
| 34 | TCC | 22233500 | 1.55 | GAA | 81910 | 1.43 | ATG | 4161780 | 1.62 | TCC | 9110400 | 1.64 | CCT | 7339790 | 1.46 | CTG | 1606870 | 1.42 |
| 35 | GTG | 21853000 | 1.52 | ATG | 80182 | 1.4 | CAA | 4102950 | 1.59 | GTG | 8982220 | 1.61 | CTC | 7080790 | 1.41 | TGG | 1360310 | 1.2 |
| 36 | CAC | 21829700 | 1.52 | CAT | 79957 | 1.4 | TTG | 4087270 | 1.59 | CAC | 8952730 | 1.61 | GAG | 7078150 | 1.41 | CCA | 1358590 | 1.2 |
| 37 | GTT | 21724100 | 1.51 | TTT | 78985 | 1.38 | ACT | 3931880 | 1.53 | GCT | 8825050 | 1.58 | GAT | 7008680 | 1.4 | AGG | 1326960 | 1.17 |
| 38 | AAC | 21690900 | 1.51 | TTG | 78186 | 1.37 | AGT | 3926010 | 1.53 | AGC | 8813760 | 1.58 | ATC | 7003110 | 1.4 | CCT | 1322460 | 1.17 |
| 39 | GCT | 21274300 | 1.48 | AAA | 77209 | 1.35 | ACC | 3706080 | 1.44 | TGC | 8484090 | 1.52 | GGA | 6722750 | 1.34 | GAG | 1303230 | 1.15 |
| 40 | AGC | 21265000 | 1.48 | CAA | 76982 | 1.35 | GGT | 3694270 | 1.44 | GCA | 8472420 | 1.52 | TCC | 6716520 | 1.34 | CTC | 1301560 | 1.15 |
| 41 | TGC | 20857300 | 1.45 | AGT | 76931 | 1.34 | GAC | 3353980 | 1.3 | GTT | 8433190 | 1.51 | GTG | 6670040 | 1.33 | TGC | 1297610 | 1.15 |
| 42 | GCA | 20842700 | 1.45 | ACT | 76498 | 1.34 | GTC | 3349380 | 1.3 | AAC | 8400340 | 1.51 | CAC | 6663660 | 1.33 | GCA | 1296840 | 1.14 |
| 43 | TAG | 19550800 | 1.36 | GTT | 59872 | 1.05 | AAC | 3309200 | 1.29 | GGG | 7747920 | 1.39 | TGC | 6557430 | 1.31 | GTG | 1294070 | 1.14 |
| 44 | CTA | 19529800 | 1.36 | AAC | 58027 | 1.01 | AAT | 3307850 | 1.28 | CCC | 7740960 | 1.39 | GCA | 6552310 | 1.31 | CAC | 1292950 | 1.14 |
| 45 | ATC | 18762300 | 1.31 | TAG | 57698 | 1.01 | GTT | 3305540 | 1.28 | TAG | 7414170 | 1.33 | TAC | 6522730 | 1.3 | GGA | 1249090 | 1.1 |
| 46 | GAT | 18762000 | 1.31 | CTA | 57593 | 1.01 | ATT | 3300850 | 1.28 | CTA | 7398950 | 1.33 | GTA | 6521130 | 1.3 | TCC | 1246610 | 1.1 |
| 47 | CCC | 18504200 | 1.29 | ATC | 56196 | 0.98 | TAA | 3115290 | 1.21 | GAT | 7143270 | 1.28 | GCT | 6310000 | 1.26 | GCT | 1157560 | 1.02 |
| 48 | GGG | 18487300 | 1.29 | GAT | 56154 | 0.98 | TTA | 3115170 | 1.21 | ATC | 7143210 | 1.28 | AGC | 6303550 | 1.26 | AGC | 1156700 | 1.02 |
| 49 | GTA | 16801700 | 1.17 | ATT | 45437 | 0.79 | TAG | 3006610 | 1.17 | GGT | 6684130 | 1.2 | ACC | 4914590 | 0.98 | GGT | 926800 | 0.82 |
| 50 | TAC | 16788700 | 1.17 | AAT | 45156 | 0.79 | CTA | 3005750 | 1.17 | ACC | 6663790 | 1.2 | GGT | 4912290 | 0.98 | ACC | 923423 | 0.81 |
| 51 | GGT | 16312800 | 1.14 | TAC | 43811 | 0.76 | ATC | 2932710 | 1.14 | GGC | 6317210 | 1.13 | GAC | 4761640 | 0.95 | GTC | 918112 | 0.81 |
| 52 | ACC | 16303100 | 1.13 | GTA | 43646 | 0.76 | GAT | 2926850 | 1.14 | GCC | 6315270 | 1.13 | GTC | 4757780 | 0.95 | GAC | 917906 | 0.81 |
| 53 | GTC | 15418400 | 1.07 | TTA | 42764 | 0.75 | ATA | 2709020 | 1.05 | GTC | 6310360 | 1.13 | CCC | 4457980 | 0.89 | GGG | 677064 | 0.6 |
| 54 | GAC | 15417700 | 1.07 | TAA | 41957 | 0.73 | TAT | 2705230 | 1.05 | GAC | 6301900 | 1.13 | GGG | 4454370 | 0.89 | CCC | 675327 | 0.59 |
| 55 | GCC | 15121000 | 1.05 | CGG | 40900 | 0.71 | TAC | 2380320 | 0.92 | GTA | 6251220 | 1.12 | GGC | 3755240 | 0.75 | GCC | 593532 | 0.52 |
| 56 | GGC | 15114900 | 1.05 | CCG | 40772 | 0.71 | GTA | 2377420 | 0.92 | TAC | 6234830 | 1.12 | GCC | 3754670 | 0.75 | GGC | 593440 | 0.52 |
| 57 | CGT | 3697050 | 0.25 | TAT | 38253 | 0.67 | CCG | 1176230 | 0.45 | CGT | 1645700 | 0.29 | CGT | 936460 | 0.18 | CGT | 162559 | 0.14 |
| 58 | ACG | 3692550 | 0.25 | ATA | 37749 | 0.66 | CGG | 1174630 | 0.45 | ACG | 1641910 | 0.29 | ACG | 935911 | 0.18 | ACG | 161987 | 0.14 |
| 59 | CCG | 3480980 | 0.24 | CGC | 33914 | 0.59 | CGC | 936137 | 0.36 | CCG | 1535370 | 0.27 | TCG | 777200 | 0.15 | TCG | 132953 | 0.11 |
| 60 | CGG | 3479020 | 0.24 | GCG | 33581 | 0.58 | GCG | 934125 | 0.36 | CGG | 1535070 | 0.27 | CGA | 775799 | 0.15 | CGA | 132613 | 0.11 |
| 61 | TCG | 3062260 | 0.21 | ACG | 25464 | 0.44 | CGT | 927633 | 0.36 | TCG | 1349560 | 0.24 | CGG | 645858 | 0.12 | CCG | 82949 | 0.07 |
| 62 | CGA | 3058780 | 0.21 | CGT | 24700 | 0.43 | ACG | 927282 | 0.36 | CGA | 1348190 | 0.24 | CCG | 645663 | 0.12 | CGG | 82572 | 0.07 |
| 63 | CGC | 2842690 | 0.19 | CGA | 22029 | 0.38 | TCG | 780818 | 0.3 | GCG | 1263190 | 0.22 | CGC | 541039 | 0.1 | CGC | 70660 | 0.06 |
| 64 | GCG | 2841900 | 0.19 | TCG | 21731 | 0.38 | CGA | 780149 | 0.3 | CGC | 1260940 | 0.22 | GCG | 540434 | 0.1 | GCG | 70569 | 0.06 |

**Table S3 – Trinucleotide frequencies in the isochores of the chicken genome**

Trinucleotide frequencies in the chicken genome (without repeating sequences) and in the isochores L1, L2, H1, H2, H3 and H4.

|  | **Total** | | | **H4** | | | **H3** | | | **H2** | | | **H1** | | | **L2** | | | **L1** | | |
| --- | --- | --- | --- | --- | --- | --- | --- | --- | --- | --- | --- | --- | --- | --- | --- | --- | --- | --- | --- | --- | --- |
|  | triplet | amount | % | triplet | amount | % | triplet | amount | % | triplet | amount | % | triplet | amount | % | triplet | amount | % | triplet | amount | % |
| 1 | TTT | 32640000 | 3.67 | CAG | 116589 | 3.52 | CTG | 568039 | 3.33 | CAG | 3233230 | 2.99 | TTT | 9122750 | 3.37 | TTT | 14449200 | 4.01 | AAA | 5962340 | 4.56 |
| 2 | AAA | 32567200 | 3.66 | CTG | 115338 | 3.49 | CAG | 557384 | 3.27 | CTG | 3200580 | 2.96 | AAA | 9049280 | 3.35 | AAA | 14417200 | 4 | TTT | 5958760 | 4.56 |
| 3 | CTG | 21095300 | 2.37 | CCC | 111731 | 3.38 | TGC | 474303 | 2.78 | AAA | 2788020 | 2.57 | CTG | 6858310 | 2.54 | ATT | 9324260 | 2.59 | ATT | 4010070 | 3.06 |
| 4 | CAG | 21076800 | 2.37 | GGG | 109142 | 3.3 | GGG | 468359 | 2.75 | TTT | 2756560 | 2.54 | CAG | 6829650 | 2.52 | AAT | 9322660 | 2.59 | AAT | 4008550 | 3.06 |
| 5 | ATT | 20854700 | 2.34 | GCA | 97649 | 2.95 | GCA | 466992 | 2.74 | GCA | 2655950 | 2.45 | TCT | 5976970 | 2.21 | TCT | 8271470 | 2.29 | TTA | 3431790 | 2.62 |
| 6 | AAT | 20845200 | 2.34 | TGC | 96853 | 2.93 | CCC | 462901 | 2.72 | TGC | 2638110 | 2.44 | AGA | 5944530 | 2.2 | AGA | 8262520 | 2.29 | TAA | 3428080 | 2.62 |
| 7 | TCT | 19825600 | 2.23 | AGC | 94420 | 2.85 | GCT | 457052 | 2.68 | AGC | 2542770 | 2.35 | TGT | 5935200 | 2.19 | TGT | 8195210 | 2.27 | TAT | 3254310 | 2.49 |
| 8 | AGA | 19800200 | 2.22 | GCT | 92763 | 2.8 | AGC | 449745 | 2.64 | GCT | 2520940 | 2.33 | CTT | 5878210 | 2.17 | ACA | 8161200 | 2.26 | ATA | 3252260 | 2.48 |
| 9 | TGT | 19625600 | 2.2 | CCA | 83921 | 2.53 | TGG | 407685 | 2.39 | CCA | 2246480 | 2.07 | ACA | 5861200 | 2.17 | TTC | 8085790 | 2.24 | AGA | 3060090 | 2.34 |
| 10 | ACA | 19561400 | 2.2 | TGG | 81677 | 2.47 | CCA | 399370 | 2.34 | TGG | 2201790 | 2.03 | AAG | 5843140 | 2.16 | GAA | 8083390 | 2.24 | TCT | 3058850 | 2.34 |
| 11 | CTT | 19284900 | 2.16 | GCC | 80267 | 2.42 | CCT | 378367 | 2.22 | AGA | 2185870 | 2.02 | TTC | 5677760 | 2.1 | TAA | 8060730 | 2.24 | GAA | 3052240 | 2.33 |
| 12 | AAG | 19251000 | 2.16 | GGC | 79313 | 2.4 | AGG | 376498 | 2.21 | ACA | 2180500 | 2.01 | GAA | 5660570 | 2.09 | TTA | 8059740 | 2.24 | TTC | 3051380 | 2.33 |
| 13 | TTC | 19100600 | 2.14 | CAC | 78014 | 2.36 | GAG | 375014 | 2.2 | TCT | 2168160 | 2 | ATT | 5652710 | 2.09 | CTT | 8056730 | 2.23 | TGT | 3009890 | 2.3 |
| 14 | GAA | 19086400 | 2.14 | CCT | 77398 | 2.34 | GTG | 374395 | 2.2 | CCT | 2148830 | 1.98 | AAT | 5645510 | 2.09 | AAG | 8046160 | 2.23 | ACA | 3004630 | 2.29 |
| 15 | TGA | 17939400 | 2.01 | AGG | 76985 | 2.32 | CTC | 372938 | 2.19 | AGG | 2143780 | 1.98 | TGC | 5488430 | 2.03 | CTG | 7874960 | 2.18 | CTT | 2921930 | 2.23 |
| 16 | TTA | 17926300 | 2.01 | GAG | 76533 | 2.31 | CAC | 364128 | 2.14 | CTC | 2125860 | 1.96 | GCA | 5469730 | 2.02 | CAG | 7861770 | 2.18 | AAG | 2919720 | 2.23 |
| 17 | TCA | 17922000 | 2.01 | CTC | 76161 | 2.3 | GGA | 356822 | 2.09 | TGT | 2125440 | 1.96 | TGA | 5368240 | 1.98 | TGA | 7515420 | 2.08 | TGA | 2819020 | 2.15 |
| 18 | TAA | 17915000 | 2.01 | GTG | 76076 | 2.3 | TCC | 356268 | 2.09 | AAG | 2116380 | 1.95 | TCA | 5350960 | 1.98 | TCA | 7503120 | 2.08 | TCA | 2814720 | 2.15 |
| 19 | TTG | 17189500 | 1.93 | TCC | 72396 | 2.19 | GGC | 339601 | 1.99 | GAG | 2109370 | 1.95 | TTG | 5202380 | 1.92 | TAT | 7214480 | 2 | TTG | 2648500 | 2.02 |
| 20 | CAA | 17147900 | 1.92 | GGA | 71853 | 2.17 | GCC | 338859 | 1.99 | CTT | 2100410 | 1.94 | CAA | 5148240 | 1.9 | ATA | 7209300 | 2 | CAA | 2646160 | 2.02 |
| 21 | TGC | 16792300 | 1.88 | ACA | 55326 | 1.67 | TTT | 309782 | 1.82 | CAC | 2076170 | 1.92 | GCT | 5146880 | 1.9 | TTG | 7189960 | 1.99 | ATG | 2630910 | 2.01 |
| 22 | GCA | 16782200 | 1.88 | TGT | 52652 | 1.59 | TGT | 307235 | 1.8 | GTG | 2022260 | 1.87 | AGC | 5127440 | 1.89 | CAA | 7168790 | 1.99 | CAT | 2626230 | 2.01 |
| 23 | ATG | 16644100 | 1.87 | AGA | 52003 | 1.57 | AAA | 305554 | 1.79 | TCC | 2000110 | 1.84 | ATG | 4941090 | 1.83 | ATG | 6954310 | 1.93 | CAG | 2478170 | 1.89 |
| 24 | CAT | 16626900 | 1.87 | TCT | 50830 | 1.53 | TCT | 299294 | 1.75 | CCC | 1996640 | 1.84 | CAT | 4926730 | 1.82 | CAT | 6945660 | 1.93 | CTG | 2478110 | 1.89 |
| 25 | TAT | 15825600 | 1.78 | ACC | 50220 | 1.51 | ACA | 298514 | 1.75 | GAA | 1990460 | 1.84 | TTA | 4886140 | 1.8 | AGT | 6517430 | 1.81 | AGT | 2447560 | 1.87 |
| 26 | ATA | 15810500 | 1.77 | GGT | 49039 | 1.48 | AGA | 295187 | 1.73 | GGA | 1988130 | 1.83 | TAA | 4875360 | 1.8 | ACT | 6514460 | 1.81 | ACT | 2444410 | 1.87 |
| 27 | GCT | 15547000 | 1.74 | AAG | 47291 | 1.43 | CTT | 281741 | 1.65 | TTC | 1985260 | 1.83 | TGG | 4591200 | 1.7 | TGC | 6177610 | 1.71 | GTT | 2203070 | 1.68 |
| 28 | AGC | 15535500 | 1.74 | TGA | 46681 | 1.41 | AAG | 278307 | 1.63 | GGG | 1965210 | 1.81 | CCA | 4552700 | 1.68 | GCA | 6176890 | 1.71 | AAC | 2201670 | 1.68 |
| 29 | AGT | 15162800 | 1.7 | TCA | 46616 | 1.41 | TGA | 266911 | 1.56 | TCA | 1943340 | 1.79 | CCT | 4532480 | 1.67 | GTT | 5961690 | 1.65 | TGC | 1916960 | 1.46 |
| 30 | ACT | 15153300 | 1.7 | CAT | 46283 | 1.4 | TCA | 263267 | 1.54 | TGA | 1923100 | 1.77 | AGG | 4528240 | 1.67 | AAC | 5951480 | 1.65 | GCA | 1914970 | 1.46 |
| 31 | GTT | 14094200 | 1.58 | CTT | 45885 | 1.38 | ATG | 260232 | 1.52 | CAA | 1893530 | 1.75 | AGT | 4487230 | 1.66 | GCT | 5632180 | 1.56 | GTA | 1892750 | 1.44 |
| 32 | AAC | 14070800 | 1.58 | ATG | 45787 | 1.38 | TTC | 258349 | 1.51 | TTG | 1854440 | 1.71 | ACT | 4472420 | 1.65 | AGC | 5628480 | 1.56 | TAC | 1892490 | 1.44 |
| 33 | TGG | 14031900 | 1.57 | AAA | 44784 | 1.35 | CAT | 258007 | 1.51 | CAT | 1824030 | 1.68 | GAG | 4397420 | 1.62 | TGG | 5152820 | 1.43 | GAT | 1871360 | 1.43 |
| 34 | CCA | 14013400 | 1.57 | TTT | 42941 | 1.29 | GAA | 257282 | 1.51 | ATG | 1811810 | 1.67 | CTC | 4391820 | 1.62 | AGG | 5145010 | 1.43 | ATC | 1867080 | 1.42 |
| 35 | CCT | 13890200 | 1.56 | CAA | 42577 | 1.28 | TTG | 253553 | 1.49 | AAT | 1665430 | 1.54 | GTG | 4384990 | 1.62 | CCT | 5144140 | 1.43 | TAG | 1855780 | 1.42 |
| 36 | AGG | 13882400 | 1.56 | GAA | 42483 | 1.28 | CAA | 248579 | 1.46 | ATT | 1664500 | 1.53 | CAC | 4333710 | 1.6 | CCA | 5137090 | 1.42 | CTA | 1853840 | 1.41 |
| 37 | GAG | 13501700 | 1.51 | TTC | 42089 | 1.27 | GGT | 241929 | 1.42 | GCC | 1622830 | 1.5 | GTT | 4240210 | 1.57 | GTG | 5032040 | 1.39 | GCT | 1697200 | 1.29 |
| 38 | CTC | 13496100 | 1.51 | CCG | 41550 | 1.25 | ACC | 237250 | 1.39 | GGC | 1612230 | 1.49 | AAC | 4200510 | 1.55 | CAC | 5012120 | 1.39 | AGC | 1692610 | 1.29 |
| 39 | GTG | 13481700 | 1.51 | CGG | 41547 | 1.25 | GTT | 194123 | 1.14 | ACT | 1497380 | 1.38 | GGA | 4149230 | 1.53 | GAG | 4979320 | 1.38 | AGG | 1611860 | 1.23 |
| 40 | CAC | 13448600 | 1.51 | TTG | 40661 | 1.23 | AGT | 193440 | 1.13 | AAC | 1493960 | 1.38 | TCC | 4144260 | 1.53 | CTC | 4968080 | 1.38 | CCT | 1608950 | 1.23 |
| 41 | GGA | 12864100 | 1.44 | CGC | 36346 | 1.09 | ACT | 191790 | 1.12 | AGT | 1484260 | 1.37 | TAT | 4123240 | 1.52 | GAT | 4928910 | 1.37 | TGG | 1596720 | 1.22 |
| 42 | TCC | 12864000 | 1.44 | GCG | 36244 | 1.09 | AAC | 191074 | 1.12 | GTT | 1464360 | 1.35 | ATA | 4112940 | 1.52 | ATC | 4923760 | 1.36 | CCA | 1593800 | 1.22 |
| 43 | GAT | 11862200 | 1.33 | GAC | 35344 | 1.06 | GAT | 189666 | 1.11 | TAA | 1390090 | 1.28 | GAT | 3527450 | 1.3 | GTA | 4852720 | 1.34 | GTG | 1591970 | 1.21 |
| 44 | ATC | 11850500 | 1.33 | GTC | 34611 | 1.04 | ATC | 188275 | 1.1 | TTA | 1387760 | 1.28 | ATC | 3516130 | 1.3 | TAC | 4842660 | 1.34 | CAC | 1584440 | 1.21 |
| 45 | GTA | 10970500 | 1.23 | ATC | 33871 | 1.02 | AAT | 177720 | 1.04 | ACC | 1347410 | 1.24 | GTA | 3155230 | 1.16 | GGA | 4770530 | 1.32 | GAG | 1564080 | 1.19 |
| 46 | TAC | 10954700 | 1.23 | GAT | 33419 | 1.01 | ATT | 177659 | 1.04 | ATC | 1321340 | 1.22 | TAC | 3138560 | 1.16 | TCC | 4766010 | 1.32 | CTC | 1561210 | 1.19 |
| 47 | TAG | 10565900 | 1.18 | AGT | 32908 | 0.99 | GTC | 175026 | 1.02 | GGT | 1318050 | 1.21 | GGG | 3094190 | 1.14 | TAG | 4673680 | 1.29 | GGA | 1527480 | 1.16 |
| 48 | CTA | 10553700 | 1.18 | ACT | 32838 | 0.99 | GAC | 173207 | 1.01 | GAT | 1311420 | 1.21 | CCC | 3084760 | 1.14 | CTA | 4669600 | 1.29 | TCC | 1524940 | 1.16 |
| 49 | CCC | 9284290 | 1.04 | AAC | 32078 | 0.97 | CCG | 154820 | 0.91 | ATA | 1109300 | 1.02 | TAG | 3018950 | 1.11 | GGT | 3337410 | 0.92 | GTC | 1084220 | 0.82 |
| 50 | GGG | 9276200 | 1.04 | GTT | 30781 | 0.93 | CGG | 154253 | 0.9 | TAT | 1106010 | 1.02 | CTA | 3001800 | 1.11 | ACC | 3329460 | 0.92 | GAC | 1083240 | 0.82 |
| 51 | GGT | 8884710 | 0.99 | ATT | 25489 | 0.77 | TTA | 141486 | 0.83 | GAC | 1056920 | 0.97 | GGT | 2883030 | 1.06 | GTC | 3183810 | 0.88 | GGT | 1055250 | 0.8 |
| 52 | ACC | 8877020 | 0.99 | AAT | 25357 | 0.76 | TAA | 141053 | 0.82 | GTC | 1048470 | 0.96 | GGC | 2862410 | 1.06 | GAC | 3180210 | 0.88 | ACC | 1054870 | 0.8 |
| 53 | GCC | 8420220 | 0.94 | ACG | 25302 | 0.76 | GCG | 136218 | 0.8 | TAC | 951584 | 0.88 | GCC | 2859110 | 1.05 | GGG | 2873430 | 0.79 | GGG | 765867 | 0.58 |
| 54 | GGC | 8418560 | 0.94 | CGT | 24840 | 0.75 | CGC | 135477 | 0.79 | GTA | 940004 | 0.86 | ACC | 2857810 | 1.05 | CCC | 2862930 | 0.79 | CCC | 765329 | 0.58 |
| 55 | GTC | 8065780 | 0.9 | TAA | 19645 | 0.59 | CGT | 112806 | 0.66 | CTA | 908309 | 0.84 | GTC | 2539650 | 0.94 | GGC | 2781570 | 0.77 | GGC | 743431 | 0.56 |
| 56 | GAC | 8056710 | 0.9 | TTA | 19393 | 0.58 | ACG | 112020 | 0.65 | TAG | 897668 | 0.83 | GAC | 2527780 | 0.93 | GCC | 2778010 | 0.77 | GCC | 741149 | 0.56 |
| 57 | ACG | 2923610 | 0.32 | TAC | 18255 | 0.55 | TAT | 111967 | 0.65 | CCG | 575604 | 0.53 | CGT | 1012710 | 0.37 | ACG | 973652 | 0.27 | CGT | 253500 | 0.19 |
| 58 | CGT | 2923400 | 0.32 | GTA | 17969 | 0.54 | GTA | 111793 | 0.65 | CGG | 573477 | 0.53 | ACG | 1007210 | 0.37 | CGT | 972301 | 0.27 | ACG | 252245 | 0.19 |
| 59 | CCG | 2262920 | 0.25 | CGA | 17287 | 0.52 | ATA | 111153 | 0.65 | ACG | 553184 | 0.51 | CCG | 777149 | 0.28 | CGA | 614950 | 0.17 | TCG | 159739 | 0.12 |
| 60 | CGG | 2258040 | 0.25 | TCG | 16924 | 0.51 | TAC | 111110 | 0.65 | CGT | 547250 | 0.5 | CGG | 776158 | 0.28 | TCG | 612355 | 0.17 | CGA | 158729 | 0.12 |
| 61 | CGC | 2198490 | 0.24 | CTA | 16459 | 0.49 | TAG | 104009 | 0.61 | CGC | 536560 | 0.49 | GCG | 770834 | 0.28 | GCG | 603225 | 0.16 | CCG | 119447 | 0.09 |
| 62 | GCG | 2195350 | 0.24 | TAG | 15858 | 0.47 | CTA | 103695 | 0.6 | GCG | 530099 | 0.49 | CGC | 768347 | 0.28 | CGC | 602787 | 0.16 | CGC | 118972 | 0.09 |
| 63 | CGA | 1886070 | 0.21 | TAT | 15610 | 0.47 | CGA | 76350 | 0.44 | CGA | 370658 | 0.34 | TCG | 650146 | 0.24 | CCG | 594345 | 0.16 | CGG | 118941 | 0.09 |
| 64 | TCG | 1884090 | 0.21 | ATA | 15535 | 0.47 | TCG | 75799 | 0.44 | TCG | 369128 | 0.34 | CGA | 648099 | 0.24 | CGG | 593667 | 0.16 | GCG | 118730 | 0.09 |
